# Preliminary screening of the possible protective effect of Moroccan propolis against chromium-induced nephrotoxicity in animal model

**DOI:** 10.14202/vetworld.2020.1327-1333

**Published:** 2020-07-14

**Authors:** Soukaina El-Guendouz, Soumia Zizi, Youssef Elamine, Badiaa Lyoussi

**Affiliations:** Laboratory of Physiology-Pharmacology and Environmental Health, Faculty of Sciences Dhar-Mahraz, University Sidi Mohamed Ben Abdellah, Fez, Morocco

**Keywords:** antioxidant, chromium, phenols, propolis, renal damage

## Abstract

**Background and Aim:**

Hexavalent chromium (Cr (VI)) compounds have been shown to induce nephrotoxicity associated with oxidative stress in humans and animals. The aim of the present study was to investigate the nephroprotective effect of bee propolis, as highly antioxidant natural product, *in vivo* using an animal model.

**Materials and Methods:**

First of all, total phenol and flavonoid contents of propolis sample were estimated *in vitro*. Afterward, to study the protective effect of propolis on renal damages caused by an injection of a single dose of potassium dichromate (15 mg/kg b.wt), 24 male Wister rats were divided into test and control groups. Propolis treatment was performed by oral gavage of 100 mg/kg b.wt/day, while the control groups received water instead. The 24 h urine was collected and blood samples were withdrawn before and after each treatment for further analysis.

**Results:**

Propolis revealed to be rich in polyphenols and flavonoids. Chromate provoked a nephrotoxic effect expressed by a drastic decrease in glomerular filtration assessed by creatinine clearance. However, the administration of propolis attenuated the renal damages induced by the chromate. This attenuation can be seen by the increase of creatinine clearance when comparing propolis treated group to the non-treated group.

**Conclusion:**

Propolis showed a protective potential against chromate-induced nephrotoxicity through the amelioration of chromate’s toxic effects. It might be concluded that propolis could be effective as chemoprotectant in the management of potassium dichromate-induced nephrotoxicity.

## Introduction

The biotoxic effects of many heavy metals such as arsenic, barium, cadmium, chromium (Cr), lead, and selenium on human health are of great concern. Those metals originate from various sources comprising mining, natural weathering of the earth’s crust, sewage discharge, industrial effluents, and many others. In general, exposure to those toxicants can be by ingestion, inhalation, or skin contact. Cr, a plentiful metal in the earth’s crust, this dangerous element exists in the environment at different degrees of oxidation ranging from (+2) to (+6). However, the most common oxidation states of Cr are trivalent Cr^+3^ and hexavalent Cr^+6^ [1]. It was reported that both states (Cr^+3^ and Cr^+6^) are toxic to humans, animals, and plants [2-4]. Naturally occurring Cr compounds are generally in the trivalent state, while hexavalent Cr compounds (the most toxic form) are mostly derived from human activities [5]. According to Jaishankar *et al*. [6], Cr^+3^ has low membrane permeability, which makes it generally innocuous comparing to Cr^+6^. The toxicity of this last form is more hazardous because it penetrates the cells much more easily than does the Cr^+3^ [6], while, Cr^+3^, in trace concentrations, is essential for regular operation of human vascular and metabolic systems as well as combating diabetes in trace. Contrariwise Cr^+6^ may cause health problems including allergic reactions, skin rash, nose irritations and nosebleed, ulcers, immune system deterioration, genetic material alteration, and kidney and liver damage and may even go as far as death of the individual [7,8].

Moreover, Cr exposure has been associated with kidney diseases, according to the urinary data, these kidney toxicants seem to target mainly the proximal tubular cells leading to oxidative stress, endoplasmic reticulum stress, and mitochondrial damages resulting in apoptosis and necrosis [7,8]. On the other hand, nowadays, natural products provide an excellent strategy toward identifying new bioactive compounds for combating a large number of human diseases, especially those induced by oxidative stress. Propolis is a resinous substance collected by honeybees from parts of plants, buds, and exudates and mixed with bees wax and salivary enzymes. It is mostly constituted by resin, wax, essential oils, and pollen. Its richness in bioactive polyphenols makes it in a suitable natural product for the present contribution. The chemical variability found in propolis can be attributed to the geographical location, collection site, botanical origin, bee species, and the climate [9,10]. Different studies have shown that propolis has a broad spectrum of biological properties, such as antimicrobial, anti-inflammatory, antioxidant, antidiabetic, spasmolytic, anesthetic, anticancer, and immunomodulatory effects [9-15].

To the best of our knowledge, the protective effect of propolis against K_2_Cr_2_O_7_-induced nephrotoxicity has not been explored. The aim of this work was to investigate whether using propolis as a natural product, can prevent or improve the effects of Cr-induced nephrotoxicity in an animal model.

## Materials and Methods

### Ethical approval

The ethical approval was obtained from Sidi Mohamed Ben Abdellah University in Fez, under the responsibility of the Animal Facility and the Laboratory of Physiology-Pharmacology and Environmental Health, Faculty of Sciences Dhar EL Mahraz of Fez, Morocco (USMBA-PPSE 2016-05). The care and handling of the animals were in accordance with the internationally accepted standard guidelines for the Care and Use of Laboratory Animals, and the protocol was approved by our institutional committee on animal care, University Sidi Mohamed Ben Abdellah, Faculty of Sciences Dhar EL Mahraz Fez, Morocco. All efforts were made to minimize animal suffering and the number of animals used.

###  Study period and location

The experiment was conducted at the Laboratory of Physiology-Pharmacology and Environmental Health, Faculty of Sciences Dhar EL Mahraz Fez, Morocco, during March to May 2017.

### Sample preparation

Propolis sample was obtained from a beekeeper’s association in Fez, Morocco. Ten milliliters of distilled water were added to 100 mg of propolis powder for total phenols and flavonoids determination. Regarding the *in vivo* experiment, distilled water was added to obtain the required propolis concentration which was orally administered to the animals for 10 days.

### Determination of phenolic groups

#### Total phenol content

The total polyphenol content in propolis sample was determined using the method of El-Guendouz *et al*. [10]. Propolis (25 μL) was mixed with 125 μL of Folin–Ciocalteu reagent (0.2 N) and 100 μL of 7.5% Na_2_CO_3_, and the absorbance was measured at 760 nm after 2 h of incubation at room temperature. The total polyphenol content was expressed as milligrams of ferulic acid equivalents per milliliter (mg FAE/mL) using a calibration curve.

### Total flavones/flavonols content

The amount of flavones and flavonols in the propolis was determined according to the method of El-Guendouz *et al*. [10]. Briefly, 100 μL of Al_2_Cl_3_ (20%) were added to 100 μL of extract, and after 1 h of incubation at room temperature, the absorbance was recorded at 420 nm. Total flavone and the flavonol contents were calculated as quercetin equivalents per milliliter (mg QE/mL) using a calibration curve.

### Total flavanones and dihydroflavonol contents

The quantification of total flavanone and dihydroflavonol compounds was performed according to El-Guendouz *et al*. [10], where 75 μL of sample or standard (Eriodictyol) and 2 mL of 2,4-dinitrophenylhydrazine (DNP) solution (1 g DNP in 2 mL 96% sulfuric acid, diluted to 100 mL with methanol) were mixed and heated at 50°C for 50 min. After cooling at room temperature, the mixture was diluted to 10 mL with methanolic KOH solution (10%, w/v); then, 1 mL of the resulting solution was diluted to 50 mL with methanol and the absorbance was measured at 486 nm.

### Experimental animals

Adult male Wistar rat’s weighing 190±20 g were obtained from our Animal House Breeding Center, Department of Biology, Faculty of Sciences Dhar El Mahrez Fez, Morocco. The animals were housed under standard environmental conditions (25±2°C, 55±5% humidity and 12 h/12 h light/dark cycle) and allowed free access to tap water and standard laboratory rat chow.

### Experimental design

The rats were divided into four groups of six rats each, as follows:

Group I: Served as healthy control and received distilled water (10 mL/kg b.wt/day)

Group II: Served as healthy group treated with propolis (100 mg/kg b.wt/day)

Group III: Rats of this group received distilled water (10 mL/kg b.wt/day) and were injected with potassium dichromate (15 mg/kg b.wt/single dose)

Group IV: Received propolis (100 mg/kg b.wt/day) and were injected with 15 mg/kg b.wt of potassium dichromate.

The animals were kept in metabolic cages, experimentation was continued for 10 days, and the animals received the treatments daily. Potassium dichromate aqueous solution was administered by subcutaneous injection of single dose at day 4 of the experiment and propolis was given by oral gavage daily.

### Biological mediums sampling and analysis

The 24 h urine samples were collected in graduated cylinders, to determine the urine volume for each rat, filtered, centrifuged, and stored at −20°C until the day of analysis. Blood samples were withdrawn at the beginning, before starting the experimental protocol, and after 10 days, centrifuged at 4000 r.p.m for 10 min, and then, the plasma was collected and stored at −20°C until analysis.

Plasma and urine electrolytes concentration, glucose, triglycerides, and cholesterol profiles were measured by flame spectrophotometry. Concentration of creatinine in plasma and urine was determined by the Jaffe alkaline picrate method, and creatinine concentration was measured by spectrophotometer at 500 nm. Creatinine clearance was used to evaluate the renal function, or more exactly, the glomerular filtration rate, and was calculated from plasma and urinary creatinine levels following the formula:

(Creatinine_Urine_/Creatinine_Serum_) × (Volume_Urine_ (mL)/Time (h) × 60)

### Statistical analysis

All data were analyzed using GraphPad Prism 5 (GraphPad Software, La Jolla, CA, USA) and the results were expressed as mean±standard deviation. Within group, means comparison was performed through the analysis of variance test. A significant difference between the control and experimental groups was assessed using student’s t-test. p<0.05 was considered to be statistically significant.

## Results

### Phenols

**Figure-1:**
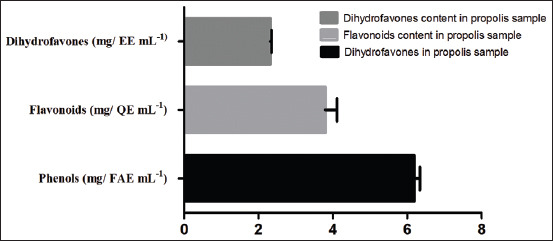
Concentration of total phenols, flavonols, flavones, flavanones, and dihydroflavonols in propolis sample.

The concentrations of total phenols, flavones and flavonols, and flavanones and dihydroflavonols found in propolis samples used in this work are displayed in Figure-1. Propolis showed a high amount of phenolic compounds with a value of 6.21±0.02 mg FAE/mL. Regarding the flavones and flavonols content, it was high as 3.91±0.20 mg QE/mL, while the amount of flavanones and dihydroflavonols amount was 2.31±0.01 mg QE/mL in the studied propolis sample (Figure-1).

### Nephrotoxicity induction and chemoprotective effect of propolis

#### Urinary volume

**Figure-2:**
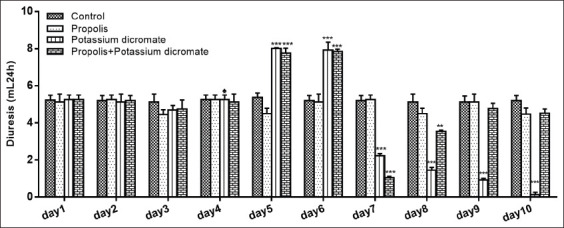
Average of urinary volume excretion (UV) of control and treated rats during 10 days of treatment. The volume of 24 h urine was measured and reported as mean±standard error of the mean for six rats in each group. ♠ Chromium injection day.

In the present work, we evaluate the effect of propolis on alleviating the chromate-induced nephrotoxicity in Wister rats after single subcutaneous dose injection. The results displayed in Figure-2, show that the urinary volume increases significantly (p<0.001) in the 5^th^ day (8.01±0.12 mL; 7.76±0.15 mL) and 6^th^ day (7.92±0.12 mL; 7.85±0.15 mL) for the none treated nephrotoxic group and for that injected with dichromate and treated with propolis, respectively, as compared to the healthy control group. Afterward, for the none treated nephrotoxic animals, a significant decrease (p<0.001) of the urine volume was observed starting from day 7 (2.22±0.25 mL) until the end of the experiment (0.12±0.10 mL). Regarding the nephrotoxic group treated with propolis, a significant decrease (p<0.001) of the diuresis on day 7 (1.05±0.38 mL) was observed. However, this diuresis starts recovering the normal volume on day 8. It is worth mentioning that the administration of propolis to healthy rats did not affect their 24 h urinary volumes (Figure-2).

#### Electrolytes levels and creatinine clearance

The effects of the interventions with propolis along with/without Cr on urinary and plasma sodium and potassium are summarized in Table-1. The results showed that significant changes can be seen in some electrolytes following Cr injection. While, no significant changes in unary Na^+^ and K^+^ were induced for the healthy group treated with propolis, as compared with control made of healthy animals. Cr injection in the absence of any treatment induced a significant decrease (p<0.001) of both parameters (Table-1). However, propolis supplementation attenuated the decrease observed following Cr injection.

**Table-1:** Concentrations of urinary and plasma electrolyte in control and treated rats after 10 days of treatment.

Groups	Urinary electrolyte concentration		Plasma electrolyte concentration	
	
	Na^+^ (mmol/L)	K^+^ (mmol/L)	Na^+^ (mmol/L)	K^+^ (mmol/L)
Control	75.25±1.47	54.6±0.75	141.00±0.70	3.1±0.81
Propolis	76.30±0.47	49.25±0.24	139.00±0.70	3.08±0.11
Potassium dichromate	22.02±1.01***	7.4±0,18***	136.00±1.41*	7.00±0.5***
Propolis/potassium dichromate	58.80±0.15*	35.56±0,18*	127.00±1.58*	4.17±0.14*

Data are presented as mean±SD, **p<0.05 compared to control, **p<0.01 compared to control,

*** p<0.001 compared to control. SD: Standard deviation

Regarding the plasma electrolyte Na^+^, both nephrotoxic groups revealed a significant difference (p<0.05) as compared to the control healthy group. While, the plasmatic K^+^ has significantly increased (p<0.001) in the none treated nephrotoxic group (7.00±0.5 mmol/L) as compared to the control (3.1±0.81 mmol/L) (Table-1). However, propolis treatment maintained this parameter around the normal values (4.17±0.14 mmol/L).

**Figure-3:**
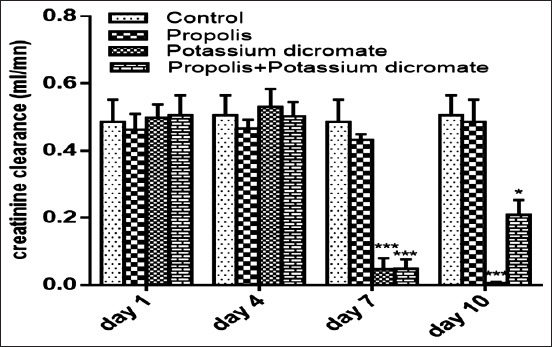
Effect of propolis and potassium dichromate on creatinine clearance in normal Wistar rats treated and control. Data are presented as mean±standard deviation, **p<0.05 compared to control, **p<0.01 compared to control, ***p<0.001 compared to control.

On the other hand, according to Figure-3, the administration of propolis to healthy rats did not affect the creatinine clearance during the experiment comparing with the control. In contrast, the injection of chromate caused a drastic reduction of renal function. This reduction can be seen by the significant decrease (p<0.001) of the creatinine clearance by the end of the experiment. Regarding the group supplemented with propolis, a significant alleviation against the adverse effect of Cr injection was observed (Figure-3).

### Osmolality and the clearance of free water

All groups showed no significant change in plasma osmolality as compared to the control. In contrast, the injection of dichromate alone decreased significantly free water clearance which provides more evidence about the occurrence of renal function disorder as compared to control group. As for the other parameters, propolis administration restored significantly (p<0.01) the renal function through maintaining these parameters in normal values (Table-2).

**Table-2:** Concentrations of biochemical parameters in control and treated rats after 10 days of treatment.

Groups	U_osm (mosm/kgH2O)_	P_osm (mosm/kgH2O)_	C_osm_ _(μl/min)_	C_H20__(μl/min)_
Control	1580±9	286±6	27.62±2	−22.6±2
Propolis	1526±9	278±6	24.7±2.2	−20.20±
Potassium dichromate	440±9***	227±8	3.5±0.5***	−1.6±0.1***
Propolis/potassium dichromate	1880±9	254±6	14.4±1.2**	−11.30±**

Data are presented as mean±SD,

** p<0.05 compared to control, **p<0.01 compared to control,

*** p<0.001 compared to control. SD: Standard deviation

### Blood glucose, triglycerides, cholesterol levels, and creatinine clearance

The results of biochemical parameters showed that the dichromate leads to a significant increase in blood glucose concentrations, triglycerides, and cholesterol levels (p<0.001; p<0.001; and p<0.01, respectively). The values of those parameters increased from 1.15±0.14 g/L, 0.95±0.22 g/L, and 0.47±0.08 g/L in normal rats to 2.78±0.10 g/L, 1.34±0.03 g/L, and 1.23±0.01 g/L, respectively, in nephrotoxic individuals. The supplementation with propolis moderated the acute effect of chromate on the mentioned parameters and the values were 1.81±0.16 g/L, 1.02±0.02 g/L, and 0.61±0.12 g/L, respectively (Table-3).

**Table-3:** Effects of oral administration of propolis, potassium dichromate, propolis/potassium dichromate, and distilled water on various parameters measured (mean±SD).

Groups	Glucose (g/L)	Triglycerides (g/L)	Cholesterol (g/L)
Control	1.15±0.14	0.95±0.22	0.47±0.08
Propolis	0.85±0.12**	0.75±0.11	0.51±0.05
Potassium dichromate	2.78±0.10***	1.34±0.03***	1.23±0.01**
Propolis/potassium dichromate	1.81±0.16*	1.02±0.02**	0.61±0.12*

** p<0.05 compared to control, **p<0.01 compared to control,

*** p<0.001 compared to control. SD: Standard deviation

## Discussion

Heavy metals toxicity in organism, including that of Cr, was associated with the generation of reactive oxygen species (ROS), leading to an oxidative stress [16]. Cr is well-known as oxidizing agent able to induce tissue damages directly. The humans and animals’ exposure to Cr^6+^ compounds induce carcinogenic, mutagenic, and teratogenic effects [6,17]. Many studies showed that oral feeding of Cr leads to a significant increase in organosomatic indices of liver, kidneys, heart, and spleen [18]. Balakrishnan *et al*. [7] reported that the administration of Cr to rats significantly reduced the antioxidant markers of kidney such as superoxide dismutase and glutathione (GSH). Moreover, it increased the peroxidation markers such as malondialdehyde. Furthermore, Velma and Tchounwou [16] found that the kidney appears to be more vulnerable and sensitive to Cr-induced toxicity than the liver.

In this context, some authors found that Cr chelating agents or antioxidants such as α-tocopherol, ascorbic acid, and GSH can ameliorate Cr -induced oxidative damage by scavenging the ROS and/or preventing biological macromolecules from oxidative injury [19-21]. Kandhare *et al*. [22] and Chetyrkin *et al*. [23] reported that L-arginine and pyridoxamine, which have antioxidant properties, significantly restored alteration in serum and urine biochemical parameters.

Several authors have shown that propolis exhibited a strong antioxidant property [9,12,24-27]. This activity was attributed to some of its bioactive compounds. In general, phenolic compounds and flavonoids have been found to be the major components of propolis, and they were reported to be the responsible of its antiradical activity [9,12,26-28]. In concordance with those authors, El-Guendouz *et al*. [10] and Miguel *et al*. [12] have found that propolis samples with higher concentrations of phenolic compounds exhibit a higher antioxidant power and anti-inflammatory properties.

In this study, total phenol contents found in the propolis sample are within the range of those generally detected in methanolic extract of Portuguese propolis, 2.93-8.76 mg pinocembrin equivalent/mL and 1.28-2.76 mg QE/mL, for total phenol and flavonoid contents, respectively [29]. Our results are in concordance with those reported for Moroccan ones, which were ranging between 0.039 and 8.864 mg FAE/mL for phenols, 0.020 and 4.320 mg QE/mL extract for the flavonoids, and 0.031 and 0.532 mg EE/mL extract for dihydroflavones [10]. Even in the absence of a deep histological study to evaluate the kidney damage caused by K_2_Cr_2_O_7_, it was evident to state the occurrence of renal function disorder given the severe decrease of the urinary volume and an acute increase of urinary and plasma electrolyte Na^+^ and K^+^, blood glucose, triglycerides, and cholesterol levels following dichromate intervention. Furthermore, a significant change in creatinine clearance, osmolality, and clearance of free water was recorded as a result of this Cr toxicity. While, after the pretreatment of rats with propolis before the Cr injection, it was observed a significative acceleration in the recovery of the previously mentioned affected parameters. In parallel, we found that chronic administration of propolis did not affect urinary volume of rats.

In sum, injection of chromate decreased the urinary output, and the glomerular filtration rate assessed by creatinine clearance as compared with the control rats. This effect can be explained by the fact that the glomerular filtration rate decreased due to the obstruction of urinary outflow. Besides, the obstruction of the urinary outflow leads to an accumulation of waste products in the blood including sodium and potassium. Those observations were proved by our results and explain the death of nephrotoxic animals by the end of the experiment. The levels of the electrolytes Na^+^ and K^+^ decreased in urine and increased in plasma for chromate injected group, while, the group of animals pretreated with propolis showed a lower increment of the electrolyte levels in urine. This finding suggests that propolis exerted a protective effect on renal damage as it alleviates the Cr effect. In this regard, we have found that propolis extract increased creatinine clearance until restoration of renal function. Many studies focused on the protective role of the antioxidant effect of propolis against the toxicity of several heavy metals. Wen *et al*. [30] reported a possible protective effect of propolis against aluminum-induced toxicity, while Masry *et al*. [31] demonstrated the same effect but against lead-induced neurotoxicity. Other researchers [32] showed the positive influence of the administration of propolis on reducing the concentration of mercury in the muscles, kidneys, and liver in animal models. In a previous study [33], our group of research has reported that this propolis sample exhibits great potential as an antioxidant agent after the *in vitro* evaluation of its ability for scavenging free radicals, azino-bis3-ethylbenzothiazoline-6-sulfonic acid, and nitric oxide and by their reducing power. Hence, we can attribute the found effect in this work to this bioactive property of propolis.

On the whole, our current results showed that propolis has been useful in preventing kidney damage caused by dichromate, suggesting that this nephroprotective effect was through its antioxidant and ROS scavenging properties through the action of flavonoids and phenolic compounds it contains.

## Conclusion

Cr injection in rats caused kidney failure and it was alleviated by propolis intervention. Propolis protected against functional alterations of the kidney. The phenolic compounds were possibly involved in its beneficial effect.

From above, we speculate that propolis by virtue of its renoprotective property ameliorates potassium dichromate-induced loss of functional integrity in kidney tissues. On the basis of this study, it should be taken into consideration that the supplementation of natural antioxidants such as propolis may act as a protective agent against the toxicity of chromates and, therefore, could be a significant intervention for future testing in kidney failure. Those results will be with a high interest and can be with a potential clinical application. However, there is a clear need to characterize the quality and quantity of the constituents of propolis and to identify the mechanism of action to confirm the present results.

## Authors’ Contributions

BL: Conceptualization and supervision. SE, YE, and SZ: Methodology, laboratory experiments, and biochemical analysis. SE: manuscript writing. YE and BL: Review of the draft. All authors read and approved the final manuscript.
